# Epigenetic silencing of the WNT antagonist Dickkopf 3 disrupts normal Wnt/β-catenin signalling and apoptosis regulation in breast cancer cells

**DOI:** 10.1111/jcmm.12099

**Published:** 2013-07-24

**Authors:** Tingxiu Xiang, Lili Li, Xuedong Yin, Lan Zhong, Weiyan Peng, Zhu Qiu, Guosheng Ren, Qian Tao

**Affiliations:** a Molecular Oncology and Epigenetics Laboratory, The First Affiliated Hospital of Chongqing Medical UniversityChongqing, China; b Cancer Epigenetics Laboratory, Department of Clinical Oncology, Sir YK Pao Center for Cancer and Li Ka Shing Institute of Health Sciences, The Chinese University of Hong Kong and CUHK Shenzhen Research InstituteShatin, Hong Kong

**Keywords:** *DKK3*, breast cancer, methylation, Wnt/β-catenin

## Abstract

Dickkopf-related protein 3 (DKK3) is an antagonist of Wnt ligand activity. Reduced DKK3 expression has been reported in various types of cancers, but its functions and related molecular mechanisms in breast tumorigenesis remain unclear. We examined the expression and promoter methylation of DKK3 in 10 breast cancer cell lines, 96 primary breast tumours, 43 paired surgical margin tissues and 16 normal breast tissues. *DKK3* was frequently silenced in breast cell lines (5/10) by promoter methylation, compared with human normal mammary epithelial cells and tissues. *DKK3* methylation was detected in 78% of breast tumour samples, whereas only rarely methylated in normal breast and surgical margin tissues, suggesting tumour-specific methylation of *DKK3* in breast cancer. Ectopic expression of DKK3 suppressed cell colony formation through inducing G0/G1 cell cycle arrest and apoptosis of breast tumour cells. DKK3 also induced changes of cell morphology, and inhibited breast tumour cell migration through reversing epithelial-mesenchymal transition (EMT) and down-regulating stem cell markers. DKK3 inhibited canonical Wnt/β-catenin signalling through mediating β-catenin translocation from nucleus to cytoplasm and membrane, along with reduced active-β-catenin, further activating non-canonical JNK signalling. Thus, our findings demonstrate that DKK3 could function as a tumour suppressor through inducing apoptosis and regulating Wnt signalling during breast tumorigenesis.

## Introduction

Constitutive activation of the Wnt signalling pathway is a hallmark of multiple human cancers. Genetic and epigenetic alterations contribute to the aberrant activation of Wnt signalling, which is further involved in cell survival, proliferation, metastasis and invasion, thus contributing to cancer initiation and progression. However, unlike other tumours, β-catenin mutations are uncommon in breast cancer [1], although other mutations including those in APC [2, 3] and Axin [4] can lead to aberrant canonical signalling, indicating epigenetic mechanism especially promoter methylation involving in the induction of abnormal Wnt signalling in breast tumorigenesis.

Wnt signalling pathway is composed of canonical Wnt/β-catenin signalling and non-canonical Wnt signalling independent of β-catenin [5]. Several Wnt antagonists have been identified including Wnt inhibitory factor 1 (WIF1), secreted frizzledrelated protein (SFRP) and the Dickkopf (DKK) families. Most of them, in addition to WNTs, have been shown to be down-regulated or silenced by promoter methylation in multiple tumours, resulting in aberrant activation of Wnt signalling in tumour cells [6–10].

The DKK family consists of a group of secreted glycoproteins, including DKK1, DKK2, DKK3, DKK4, and a unique DKK3-related protein, DKKL1 (soggy) [11]. DKK1, DKK2 and DKK4 have been found to regulate canonical Wnt/β-catenin signalling by binding with LDL-receptor-related proteins (LRP5/6), whereas DKK3 modulates Wnt signalling independent of LRPs or Wnt ligands [11–15]. *DKK3* has been found to be down-regulated or silenced by promoter CpG methylation in multiple malignancies, including acute lymphoblastic leukaemia [16], gastric [17], colon [18], hepatocellular [19], renal [20], bladder [21] and cervical [22, 23] carcinomas. Although DKK3 has been demonstrated to be frequently epigenetically silenced by promoter methylation [24–26], its biological functions and exact molecular mechanisms in breast carcinogenesis remain unclear.

In this study, we assessed the expression and promoter methylation of *DKK3* in breast cancer. We also investigated its biological functions and molecular mechanisms relevant to breast cancer. Our findings demonstrated that DKK3 regulated Wnt/β-catenin and JNK signalling, thus acting as a tumour suppressor. Its tumour-specific promoter methylation appears to be a potential biomarker for early detection of breast cancer.

## Materials and methods

### Cell lines and tumour samples

Breast tumour cell lines (BT549, MDA-MB-231, MDA-MB-435, MDA-MB-468, MCF-7, T47D, SK-BR-3, YCC-B1, YCC-B3 and ZR-75-1) were used [27]. Human mammary epithelial cell lines HMEpC (Cat. no. CA-830-05a; Applied Biosystems, Foster City, CA, USA) and HMEC were used as controls. All carcinoma cell lines were maintained in RPMI 1640 (Gibco-BRL, Karlsruhe, Germany) supplemented with 10% foetal bovine serum (FBS; PAA Laboratories, Linz, Austria), 100 U/ml penicillin and 100 μg/ml streptomycin at 37°C in a humidified atmosphere containing 5% CO_2_. HMEpC and HMEC were cultured as previously described [28]. RNA samples of human normal adult breast tissue were purchased commercially (Stratagene, La Jolla, CA, USA; Millipore Chemicon, Billerica, MA, USA and BioChain Institute, Hayward, CA, USA). DNA and RNA samples were obtained from various primary breast tumour tissues, breast tumour surgical margin tissues and normal breast tissues as described previously [29]. Fresh cancer tissues and normal breast tissues were obtained from patients who underwent primary surgery at the Surgery Department of the First Affiliated Hospital of Chongqing Medical University. Clinical and pathological data of all the participants were obtained, and their demographies are summarized in Table 2. This research was approved by the Institutional Ethics Committees of the First Affiliated Hospital of Chongqing Medical University.

### 5-aza-2′-deoxycytidine (Aza) and trichostatin A (TSA) treatment

DNA demethylation treatment of breast cancer cell lines was performed as described previously [28]. Cell lines were treated with 10 μmol/l 5-aza-2′-deoxycytidine (Sigma-Aldrich, Steinheim, Germany) for 3 days and further treated with 100 nmol/l trichostatin A (Sigma-Aldrich, Deisenheim, Germany) for additional 16 hrs.

### Nucleic acid extraction

Genomic DNA and total RNA were isolated from cell lines and tissues using DNAzol and Trizol reagents (Invitrogen, Rockville, MD, USA), respectively, according to the manufacturer's recommendations [30]. Tumour material was snap-frozen in liquid nitrogen immediately within 1/2 hr after surgery. Haematoxylin/eosin-stained sections were prepared for assessing the percentage of tumour cells where samples with only >70% tumour cells were selected. Normal breast tissues were similarly prepared. Spectrophotometry ND2000 was used to determine the concentration of DNA and RNA, and their integrity was assessed by gel electrophoresis.

### Reverse transcriptase–polymerase chain reaction

First-strand cDNA was synthesized from 1 μg of total RNA using MuLV Reverse Transcriptase (Cat. no. N8080018; ABI, Foster City, CA, USA) to a final volume of 20 μl. For RT-PCR [30], samples were assayed in a 12.5 μl reaction mixture containing 2.5 μl of cDNA. Glyceraldehyde-3- phosphate dehydrogenase (GAPDH) was used as control.

### Methylation-Specific PCR analysis of DKK3 promoter

Methylation-Specific PCR (MSP) was used to determine the DKK3 promoter methylation status, as described previously [31]. Bisulfite modified DNA was amplified by two different primer pairs specific to the unmethylated (u) and methylated (m) promoter sequences respectively. The methylation-specific primers are m3: 5′-TTTCGGGTAT CGGCGTTGTC, m4: 5′-ACTAAACCGAATTACGCTACG; The unmethylation-specific primers are u3: 5′-GTTTTTTTGGGTATTGGTGTTGTT, u4: 5′-CAACTAAACCAAATTACACTACA. PCR amplification was performed for a total of 40 cycles with an annealing temperature of 60°C and 58°C respectively. Non-methylated and methylated human DNA were used as negative and positive controls respectively. MSP products were then analysed by a 2% agarose gel containing 100 bp DNA markers (MBI Fermentas, Vilnius, Lithuania).

### Flow Cytometry analysis of cell cycle status

To assess cell cycle status, MB231 cells and BT549 cells were seeded (1 × 10^6^ cells/well) in 6-well plates and transfected with 4 μg of pMH-DKK3 or pMH empty vector using Lipofectamine™ 2000 (Invitrogen, Carlsbad, CA, USA) following the manufacturer's protocol. After 48 hrs, cells were digested using 0.1% trypsin and centrifuged at 1000 r.p.m. for 5 min. Pellets were washed with PBS and fixed in ice-cold 70% ethanol for 1 hr, and treated with 100 μl of 50 mg/l propidium iodide for 30 min. at 4°C in the dark. Annexin V-FITC/PI staining was used for apoptosis analysis. Data were analysed with the CELL Quest software (BD Biosciences, San Jose, CA, USA).

### Colony formation assay

Colony formation assays were performed as described previously [28]. Cells were plated in six-well plates and transfected with 4 μg pMH-DKK3 vectors or with an empty vector using Lipofectamine™2000 (Invitrogen, Carlsbad) according to the manufacturer's instructions. At 48 hrs after transfection, cells were collected, re-plated and selected for 2 weeks in the presence of 0.2 mg/ml G418 in BT549 cell and 1.2 mg/ml G418 in MB231 cell. Surviving colonies were counted after staining with Giemsa.

### Wound healing assay

Stably transfected cells were cultured in a six-well plate until confluent. The cell layer was carefully wounded using sterile pipette tips and washed twice with PBS, and re-suspended in fresh complete medium. After incubating for 12 and 24 hrs, cells were imaged using Nikon microscope with a low magnification 10× phase contrast objective lens. The experiments were triplicated.

### Western blot analysis

Western blot was performed on protein extracts prepared at 48 hrs after transfection of the DKK3 expression vector or empty vector. Equal amounts of protein (50 μg/lane of total extract) were separated by sodium dodecyl sulphate/polyacrylamide gel electrophoresis (SDS-PAGE) and then electrophoretically transferred onto a PVDF membrane (Bio-Rad, Hercules, CA, USA). After blocking the membrane in 5% skim milk powder dissolved in PBS for 1 hr at room temperature, the antibodies used were β-catenin, JNK, and phosphor-JNK (Abcam, Cambridge, UK), active-β-catenin (Millipore), cyclin D1, c-Myc, E-Cadherin, vimentin (Epitomics Inc., Burlingame, CA, USA), and DKK3 (R&D systems, Minneapolis, MN, USA). The samples were subsequently incubated with a 1:2,000 dilution of an HRP-conjugated goat-antimouse IgG (Promega, Madison, WI, USA) for 1 hr. Bands were visualized using an enhanced chemiluminescence kit (Amersham Pharmacia Biotech, Piscataway, NJ, USA). GAPDH was used as a control.

### Statistical analysis

Statistical analyses were performed using SPSS software version 13 (SPSS Inc., Chicago, IL, USA). Chi-square test, *t-*test, non-parameter Spearman test and Fisher's exact test were used to compare experimental methylation statuses and clinical data. Differences were considered statistically significant when *P* < 0.05.

## Results

### Expression of *DKK* family genes in breast cancer cells

**Fig. 1:**
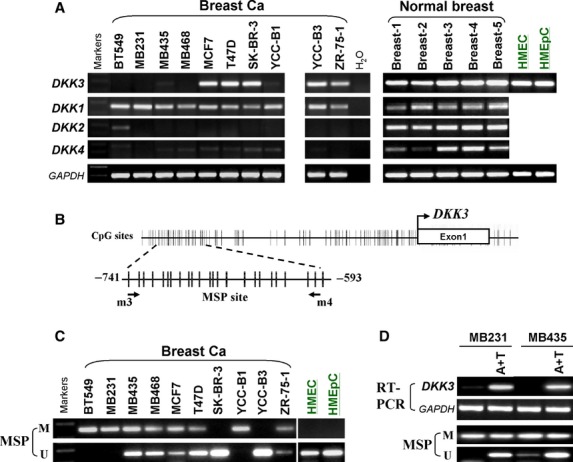
Expression and methylation status of DKKs in breast cancer cell lines and normal mammary tissues. (**A**) Expression of *DKKs* in breast cancer cell lines and human normal breast tissues detected by semiquantitative RT-PCR, with *GAPDH* as a control. (**B**) A schematic structure of the *DKK3* promoter CpG island (CGI). The white rectangle represents exon 1, and the CpG sites in the CGI are indicated with short vertical lines. The transcription start site is indicated by a curved arrow. The CGI and MSP sites (-593∼-741) are also indicated. (**C**) Methylation status of *DKK3* in breast cancer cells and normal mammary epithelial cells. (**D**) Pharmacologic demethylation and restoration of DKK3 with 5-aza-2′-deoxycytidine (Aza) and trichostatin A (TSA) treatment in MB231 and MB435 breast cancer cells. M: methylated; U: unmethylated.

We firstly examined the expression of *DKK* family genes in 10 breast cancer cell lines using semiquantitative RT-PCR. Result showed that *DKK1* was detected in all the cell lines; *DKK2* mRNA was absent in 9/10 cell lines but weakly expressed in BT549; *DKK3* was silenced/down-regulated in five cell lines (BT549, MB231, MB435, MB468 and YCC-B1); *DKK4* was significantly down-regulated in all the cell lines (Fig. 1A), while all *DKK* family genes were readily expressed in normal mammary tissues and cells. These results indicated that *DKK2*, *-3* and *-4* are frequently down-regulated in breast cancer cells.

### *DKK3* down-regulation in breast cancer correlated with its promoter methylation

We next analysed the promoters of DKK genes to evaluate whether silencing of *DKK* genes was because of promoter methylation. We found that *DKK1*, *-2* and *-3* contained typical CpG islands spanning the proximal promoter and exon regions (Fig. 1B, Figure S1), whereas no CpG island was observed in *DKK4* promoter (data not shown). We further investigated the methylation status of *DKK2* and *DKK3* promoters. MSP analysis revealed that both *DKK2* (data not shown) and *DKK3* methylation were frequently detected in breast cancer lines. Specifically, *DKK3* was methylated in eight of 10 (80%) breast cell lines with little or no expression, but not in HMEC and HMEpC cells with expression (Fig. 1C). We, thus chose to further study *DKK3* in detail.

To further clarify whether *DKK3* silencing was related to promoter methylation, cell lines treated with Aza and TSA were used. Demethylation of CpG-dinucleotides was examined by MSP analysis. Results showed that *DKK3* expression was restored in response to 5-aza-dC treatment, along with increased unmethylated promoter alleles (Fig. 1D), suggesting that promoter methylation is directly responsible for *DKK3* down-regulation in breast cancer cells.

### *DKK3* is frequently methylated in primary breast carcinomas

**Fig. 2:**
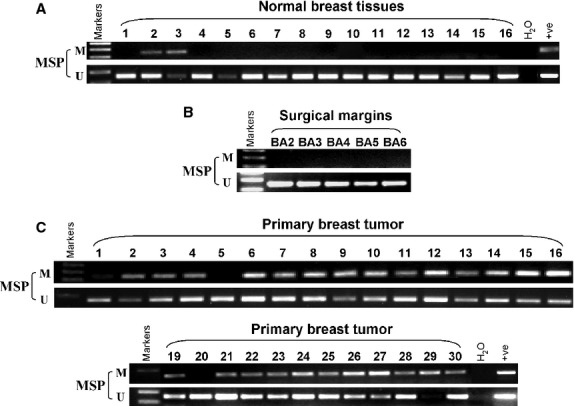
Methylation status of *DKK3* in primary breast tumour tissues. (**A**) Normal breast tissue (**B**) Breast surgical margins. (**C**) Primary breast tumour tissues. M: methylated; U: unmethylated.

To investigate *DKK3* methylation in primary breast tumour tissues, MSP analysis was employed to test 96 primary breast tumour tissues, 43 surgical marginal tissues and 16 normal breast tissues. The methylation status of *DKK3* in these three different kinds of tissues was shown in Table 1 and Figure 2. *DKK3* was methylated in 78% (75/96) of breast tumours and 12.5% of (2/16) normal breast tissues, but not in surgical margins examined (*P* < 0.05) (Fig. 2). These results indicated that *DKK3* is methylated in a virtually tumour-specific manner.

**Table 1:** Methylation status of *DKK3* promoter in primary breast tumours

Samples	*DKK3* promoter		Frequencies of methylation

	Methylated	Unmethylated	
BrCa (*n* = 96)	75	21	75/96 (78%)
BF (*n* = 34)	0	34	0/34 (0%)
BNP (*n* = 16)	2	14	2/16 (12.5%)

BrCa: breast cancer; BF: surgical margins; BNP: breast normal tissues.

We further analysed the association of *DKK3* methylation with clinicopathological features. We found that *DKK3* methylation was statistically correlated with clinical stage, lymph node metastasis and oestrogen receptor (ER) status (*P* < 0.05), but not associated with age, tumour size, progesterone receptor (PR) and hormone receptor (HR) status of breast cancer patients (Table 2).

**Table 2:** Clinicopathological characteristics and methylation status of *DKK3* in breast cancers

Clinicopathological features	Number (*n* = 96)	*DKK3* promoter methylated status		*P*-value

		Methylated	Unmethylated	
Age				
≤40	12	9	3	0.534
>40	60	49	11	
Unknown	24	17	7	
Stage				
I	6	6	0	**0.042**
II	47	40	7	
III	8	7	1	
Unknown	35	22	13	
Tumour size				
<2.0 cm	26	21	5	0.053
≥2.0 cm≤5.0 cm	36	31	5	
>5.0 cm	3	3	0	
Unknown	31	20	11	
Lymph node metastasis				
Positive	25	23	2	**0.039**
Negative	44	35	9	
Unknown	27	17	10	
Oestrogen receptor status				
Positive	47	42	5	**0.028**
Negative	21	15	6	
Unknown	28	18	10	
Progesterone receptor status				
Positive	35	30	5	0.101
Negative	33	27	6	
Unknown	28	18	10	
HR status				
Positive	37	30	7	0.194
Negative	31	27	4	
Unknown	28	18	10	

The significance of bold values are *P* < 0.05.

### DKK3 inhibits cell proliferation through inducing apoptosis in breast tumour cells

**Fig. 3:**
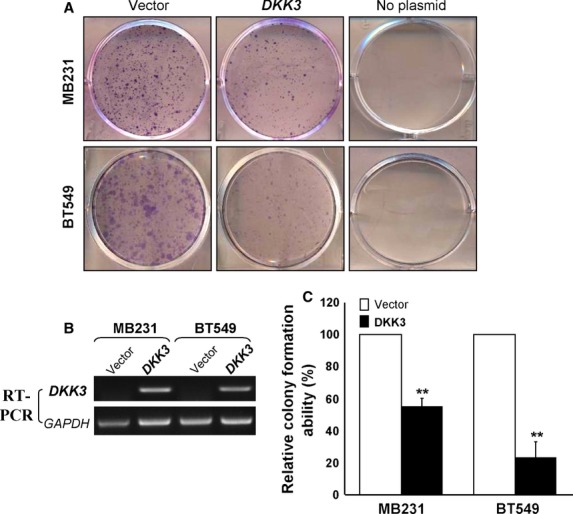
The inhibitory effect of DKK3 on colony formation assay in breast cancer. (**A**) Representative colony formation assay in vector-, and DKK3-expressing MB231 and BT549 cells. (**B**) Re-expression of *DKK3* was detected by RT-PCR. (**C**) The values are shown as the mean ± S.E. from three independent experiments (**, *P* < 0.01).

**Fig. 4:**
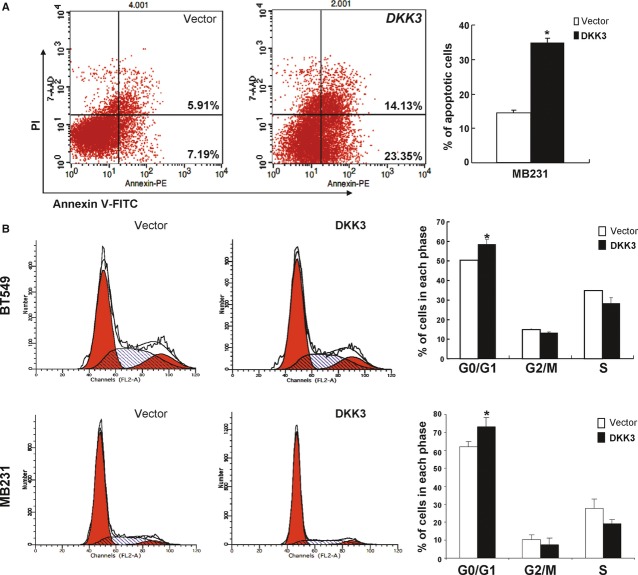
DKK3 induced apoptosis and cell cycle arrest in breast cancer. (**A**) Induction of apoptosis detected by flow cytometry with Annexin V-FITC-PI. (**B**) Effect of cell cycle distribution of vector-, DKK3-transfected MB231 and BT549 cells. Representative flow cytometry plots (left) and histograms of cell cycle alterations (right).

Silencing of *DKK3* by promoter methylation in breast cancer indicated that it may function as a TSG. To elucidate the effect of DKK3 on cell proliferation, colony formation assay was performed. Results showed ∼40–80% reduction in colony formation in DKK3-transfected MB231 and BT549 cancer cells, compared to controls (Fig. 3; *P* < 0.01). To evaluate the mechanism of DKK3 in suppressing cell proliferation, flow cytometric analyses of apoptosis and cell cycle were performed. We found that MB231 cells with DKK3 expression underwent significant apoptosis compared to controls, with 37% of cells staining positive for Annexin V (Fig. 4A). DKK3 significantly increased BT549 and MB231 cells with G0-G1 phase by 10% (*P* < 0.05), compared to controls (Fig. 4B). These results indicate that the inhibition of cell proliferation by DKK3 is most likely mediated by G0/G1 cell cycle arrest and apoptosis.

### DKK3 suppresses epithelial-mesenchymal transition and migration of breast cancer cells

**Fig. 5:**
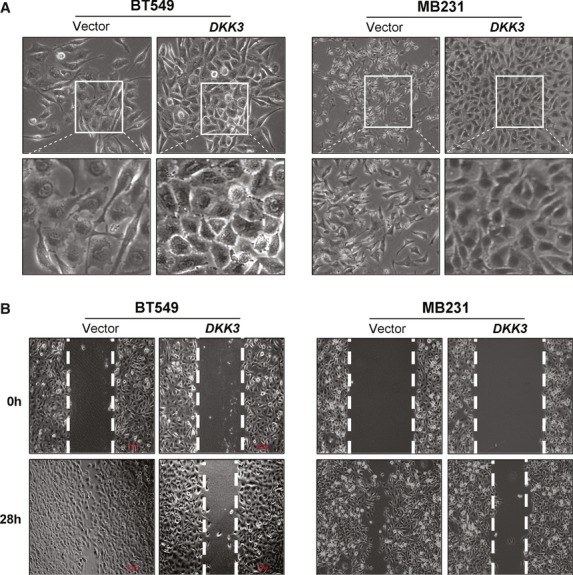
(**A**) Morphology changes of MB231 and BT549 cells transfected with DKK3 or empty vector by phase-contrast microscopy. Original magnification,×400. (**B**) The cell motilities of vector- or DKK3-transfected cells (MB231 and BT549) were tested by wound healing assay. DKK3-expressing cells spreading along the wound edges are slower compared to controls. All of the experiments were performed in triplicate.

**Fig. 6:**
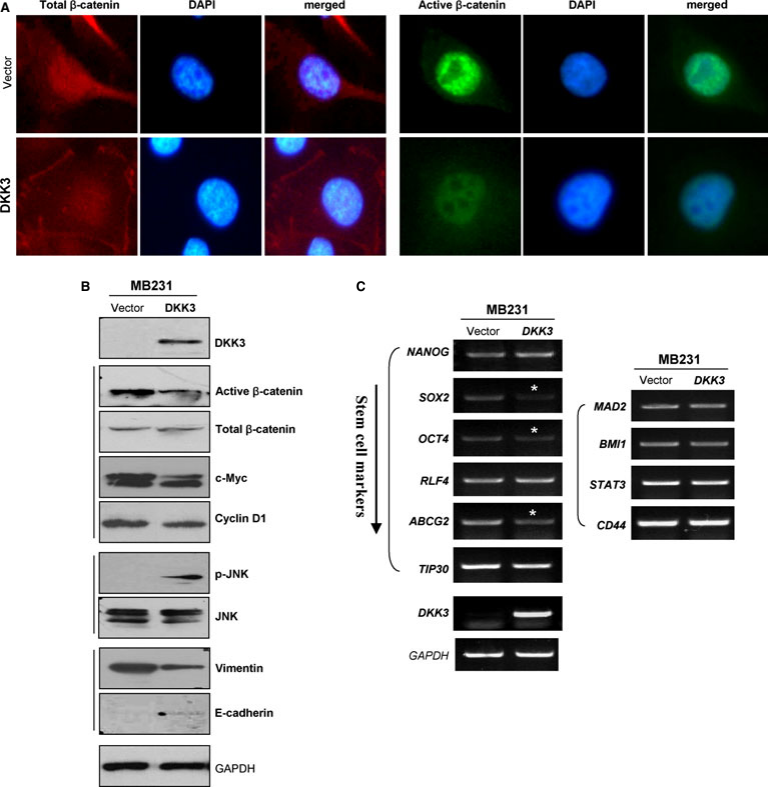
Ectopic expression of DKK3 in MB231 cells disrupted Wnt signalling. (**A**) Subcellular localization of β-catenin and active-β-catenin by immunofluorescence staining. (**B**) Western blot analysis of β-catenin, its downstream targets, JNK and EMT markers. (**C**) RT-PCR analysis of representative stem cell markers in DKK3-transfected MB231 cells. *Indicates significantly down-regulated bands.

To assess the effect of DKK3 on cell migration, we firstly examined cell morphology changes. DKK3-transfected cells regained cell–cell contacts and adherence to each other, with less aggressive behaviour, whereas the vector-transfected cells exhibited unique irregular shapes and distinguishing spread features same as the original cells (Fig. 5A), suggesting that DKK3 most likely reversed tumour cell epithelial-mesenchymal transition (EMT). Western blot analysis showed up-regulated epithelial marker E-cadherin and down-regulated mesenchymal marker vimentin in DKK3-expressing cells (Fig. 6B), indicating that DKK3 indeed negatively regulates EMT.

As EMT is implicated in regulating stem cell properties, we further investigated the expression of some stem cell-associated markers in DKK3-transfected breast cancer cells. DKK3 down-regulated stem markers such as *NANOG*, *ABCG2* and *OCT4* (Fig. 6C), thus may reverse the stem cell-like phenotype of tumour cells.

Wound healing assay was further performed to uncover the effect of DKK3 on cell migration. Results showed that DKK3-expressing cells spread along the wound edges remarkably slower than the vector-transfected cells at 28 hrs (Fig. 5B), indicating that DKK3 inhibits cell migration.

### DKK3 regulates canonical and non-canonical Wnt signalling in breast cancer cells

As Wnt/β-catenin signalling plays an important role in tumour metastasis, we thus investigated whether DKK3 counteracts this pathway for its tumour suppressive function. The effects of DKK3 on subcellular localization of β-catenin were performed in breast cancer cells with abundant level of endogenous β-catenin. Immunofluorescence staining showed increased concentration of β-catenin in cytoplasm and on the membrane in DKK3-transfected cells, compared to controls in which β-catenin was predominantly localized in nuclei. We also found significantly inhibited expression of active β-catenin and its downstream target genes, c-Myc and cyclin D1 in DKK3-expressing MB231 cells (Fig. 6A and B). Our findings suggest that DKK3 interferes with β-catenin activity and its localization, thus crippling the Wnt/β-catenin signalling pathway in breast tumorigenesis.

As JNK pathway plays an important role in non-canonical Wnt signalling, we further assessed the effect of DKK3 on JNK signalling in breast cancer cells. We found that phosphorylated JNK was increased in DKK3-expressing MB231 cells, but no obvious change was observed in the total amount of JNK protein (Fig. 6B), suggesting that DKK3 also regulates non-canonical Wnt signalling in breast tumorigenesis.

## Discussion

Wnt/β-catenin signalling is frequently implicated in various cancer development, and plays an important role in tumour initiation and progression. Aberrant DNA methylation has been shown to inactivate negative regulators of oncogenic signalling that are involved in the development and progression of human breast cancers [32]. Multiple Wnt antagonists frequently methylated in breast cancer have been identified, including *SFRP1* [33], *SFRP2* [34], *SFRP5* [35], *WIF1* [36], *DKK1* [37] and *DKK3*. The *DKK3* gene, located at 11p15.1, is a key gene in Wnt signalling pathway. Recently, *DKK3* was also reported frequently silenced as a valuable biomarker for breast cancer in the European population, but without detailed mechanistic study [24–26].

In this study, we demonstrated that *DKK3* methylation was found in 80% of breast cancer cell lines and 78% of primary breast cancer tissues in Asian population. *DKK3* was restored through demethylation with Aza and TSA in methylated breast cell lines. *DKK3* methylation was found to be associated with some clinicopathological features such as clinical stage, lymph node metastasis and ER status. Our results are in line with previous studies of *DKK3* down-regulation and methylation in a variety of cancer cell lines and tissues [24, 38–44], and its transcriptional silencing is at least partly because of aberrant hypermethylation of the promoter [16, 17, 20, 21, 25, 45–48].

We also observed frequent methylation of *DKK2* in breast cancer, which was reported in gastrointestinal and renal cancers [49]. As for *DKK4*, our findings indicate its significant down-regulation in breast cancer, however, the *DKK4* gene is devoid of a CpG island, the typical target of epigenetic regulation, thus further investigations of the molecular mechanism for DKK4 down-regulation and its biological functions are needed.

We further demonstrated the ability of DKK3 to suppress cell growth and induce apoptosis in breast cancer, supporting that it does act as a TSG in line with the findings in another report [50]. DKK3 was also shown to suppress cell migration by possibly interfering with EMT. In a previous report, no interaction was found between DKK3 and β-catenin in prostate cancer cells [51], whereas another study reported that DKK3 reduced the cytoplasmic accumulation of β-catenin in Saos-2 cells [52]. Here, we uncovered the relationship between DKK3 and Wnt/β-catenin signalling by analysing the effect of DKK3 on β-catenin. We found that DKK3 inhibited the activation of β-catenin and its downstream genes by abrogating its nuclear translocalization and activated non-canonical JNK signalling. JNK signalling mediating apoptotic cell death has been well-documented [53, 54], thus activation of JNK signalling by DKK3 may result in the induction of apoptosis in breast cancer, in line with the findings by other groups [55], although other non-canonical WNT signalling pathways may also be involved like TGF-β signalling [56].

Unlike other DKKs, the mechanism of DKK3 disrupting Wnt signalling still remains unclear, as it neither binds LRP nor Wnt ligands. Recently, two possible mechanisms have been proposed [57]: (1) Dkk3 directly interacts β-transducin repeat-containing protein (βTrCP), and further blocks the translocation of β-catenin into the nucleus [23]; (2) Dkk3 can form a protein complex with Krm on the membrane surface, thus blocks the ability of Krm proteins to inhibit Wnt signalling, which are strictly context- dependent processes upon receptors and ligands expressed [58]. Thus, further study is needed to elucidate the precise mechanisms of DKK3 interfering with Wnt signalling.

Collectively, our results demonstrate that DKK3 functions as a tumour suppressor inhibiting Wnt/β-catenin signalling in breast carcinogenesis, and raises the possibility of DKK3 methylation as a potential tumour marker and future therapeutic target for breast cancer.

## Supporting information

**Figure S1.** Schematic structures of the *DKK1* and *DKK2* promoter CpG island (CGI). The white rectangle represents exon 1, and the CpG sites in the CGI are indicated with short vertical lines. The transcription start site is indicated by a curved arrow.
