# Supplementary figures and images for: Epigenetic silencing of the WNT antagonist Dickkopf 3 disrupts normal Wnt/β-catenin signalling and apoptosis regulation in breast cancer cells
Source: J Cell Mol Med. 2013 Jul 24;17(10):1236–46. doi: 10.1111/jcmm.12099 (PMC4159020; doi:10.1111/jcmm.12099)

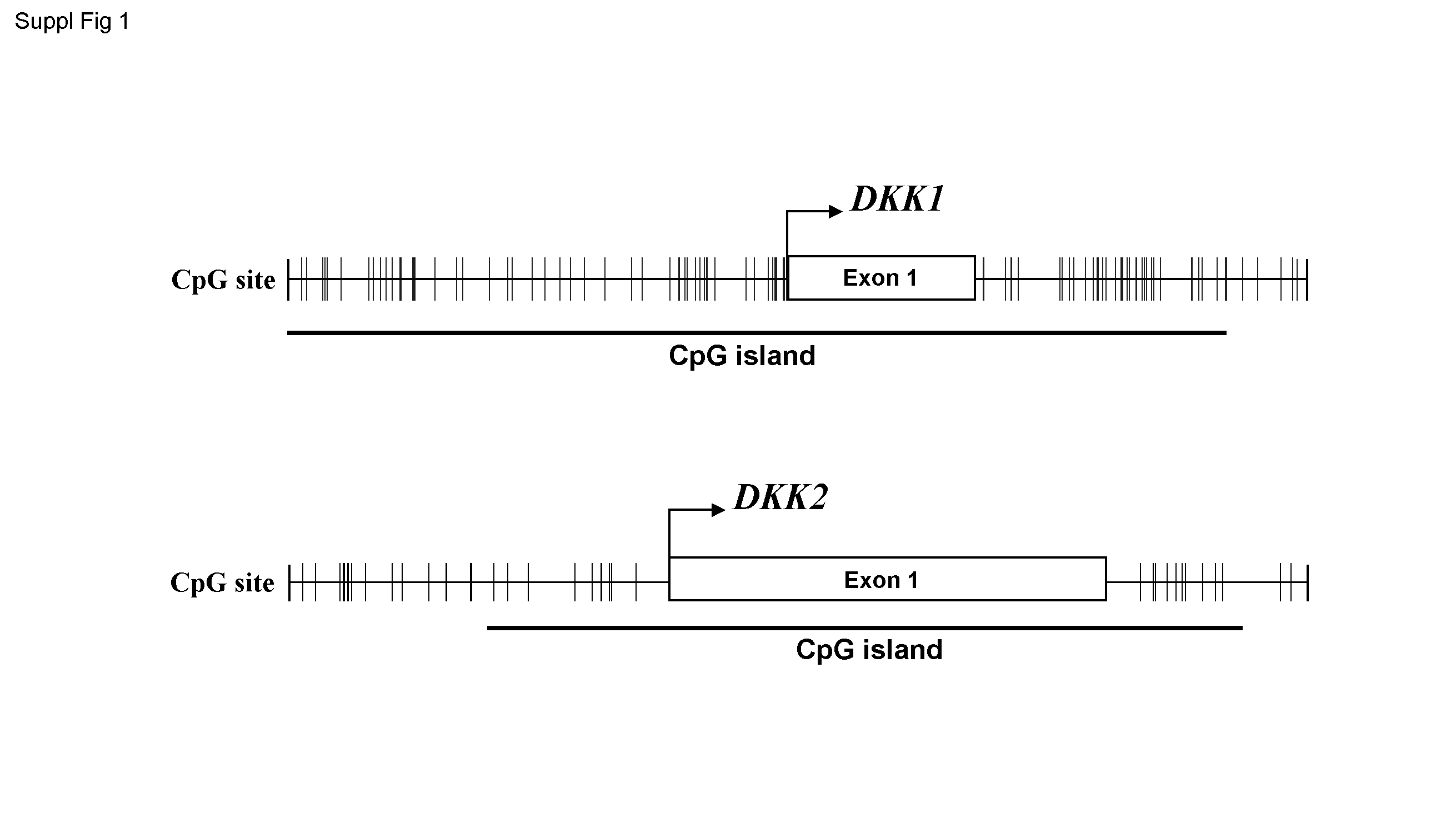

Supplement: Supplementary file 1 [file jcmm0017-1236-SD1.tif]
